# Objective consensus from decision trees

**DOI:** 10.1186/s13014-014-0270-y

**Published:** 2014-12-05

**Authors:** Paul Martin Putora, Cedric M Panje, Alexandros Papachristofilou, Alan Dal Pra, Thomas Hundsberger, Ludwig Plasswilm

**Affiliations:** Department of Radiation Oncology, Kantonsspital St. Gallen, Rorschacherstr. 95, 9007 St. Gallen, Switzerland; Department of Radiation Oncology, University Hospital Basel, Basel, Switzerland; Department of Radiation Oncology, Inselspital, University Hospital Bern, Bern, Switzerland; Department of Neurology and Haematology & Oncology, Kantonsspital St. Gallen, St. Gallen, Switzerland

**Keywords:** Consensus, Learning, Objective, Decision making, Decision tree, Algorithm

## Abstract

**Background:**

Consensus-based approaches provide an alternative to evidence-based decision making, especially in situations where high-level evidence is limited. Our aim was to demonstrate a novel source of information, objective consensus based on recommendations in decision tree format from multiple sources.

**Methods:**

Based on nine sample recommendations in decision tree format a representative analysis was performed. The most common (mode) recommendations for each eventuality (each permutation of parameters) were determined. The same procedure was applied to real clinical recommendations for primary radiotherapy for prostate cancer. Data was collected from 16 radiation oncology centres, converted into decision tree format and analyzed in order to determine the objective consensus.

**Results:**

Based on information from multiple sources in decision tree format, treatment recommendations can be assessed for every parameter combination. An objective consensus can be determined by means of mode recommendations without compromise or confrontation among the parties. In the clinical example involving prostate cancer therapy, three parameters were used with two cut-off values each (Gleason score, PSA, T-stage) resulting in a total of 27 possible combinations per decision tree. Despite significant variations among the recommendations, a mode recommendation could be found for specific combinations of parameters.

**Conclusion:**

Recommendations represented as decision trees can serve as a basis for objective consensus among multiple parties.

## Introduction

Many clinical decisions in medicine are based on formal and informal consensus agreements and recommendations [1], especially when the level of evidence is not sufficient [2]. For example, due to the lack of clinical studies many drugs used in adult medicine are not licenced for children, but may be applied in clinical routine [3]. Likewise a growing number of treatable orphan diseases, defined by their low incidence [4,5] and distinct molecular aberrations in carcinomas [6] may never be amenable to large phase III studies. The trend towards personalized medicine and limited resources is forcing us to find solutions which do not exclusively rely on classical levels of evidence as presented by Sackett et al. [7]. Even where evidence is available, this does not always translate into evidence-based practice due to lack of competency and is difficult to measure [8]. Consensus methodologies may assist us in acquiring information beyond these gaps.

Several consensus methods exist, including the Delphi process, the nominal group technique and the consensus development conference [1,9]. All of these modalities rely on discussion, negotiation, moderation and human judgement and are therefore subjective. In certain areas of medicine consensus meetings have been established as a pragmatic approach to provide guidance where evidence is not available [10-13]. In addition to “evidence-based” and “eminence-based” medicine [14], swarm-based medicine may provide guidance by extracting knowledge from the behaviour of the medical community [2]. “Crowd wisdom” can be applied in single numerical estimates as well as for combinatorial problems [15].

Learning from information of simple structure is easy. When all parties provide a numeric value a consensus can be based on simple mathematical operands like mean, median or the most common value (mode). As the structure becomes more complex consensus ceases to be intuitive. When decisions are based on patient and disease characteristics such as three different age groups (e.g. <19, 19–64, >64 years), gender (male, female) and four different histologic types (e.g. in lung cancer: adenocarcinoma, squamous cell carcinoma, small cell or “not otherwise specified”) a total of 24 eventualities arise (3 × 2 × 4). By adding further criteria the number of possible combinations rises exponentially.

Multiple decision criteria can be integrated in a guideline with the help of decision trees, such as the clinical practice guidelines of the National Comprehensive Cancer Network [16].

This study aims to demonstrate how standardised elements (diagnostic nodes) [17], can be implemented to analyse and compare multiple recommendations from various parties in order to provide an account of unbiased consensus.

## Methods

Decision trees can serve in decision support and are tree-like representations of decisions and their consequences. By connecting several elements from a starting point a decision tree can be constructed by adding possible options as branches. Recommendations (or actions) are situated at the end of the branches (analogous to leaves at the end of each branch). Nodes representing predefined parameters (diagnostic nodes [17]) were used to construct clinical decision trees. For example, the parameter gender is represented by two nodes (or branches): “male” or “female” and age e.g. by “<65 years” or “>65 years”. For later cross-comparison unified categories are important; should, for example, age be classified by one party by years (<19 years, 19 – 64 years, >64 years) and another party by category (age = old, age = young), an automated evaluation and comparison would be not possible.

**Figure 1:**
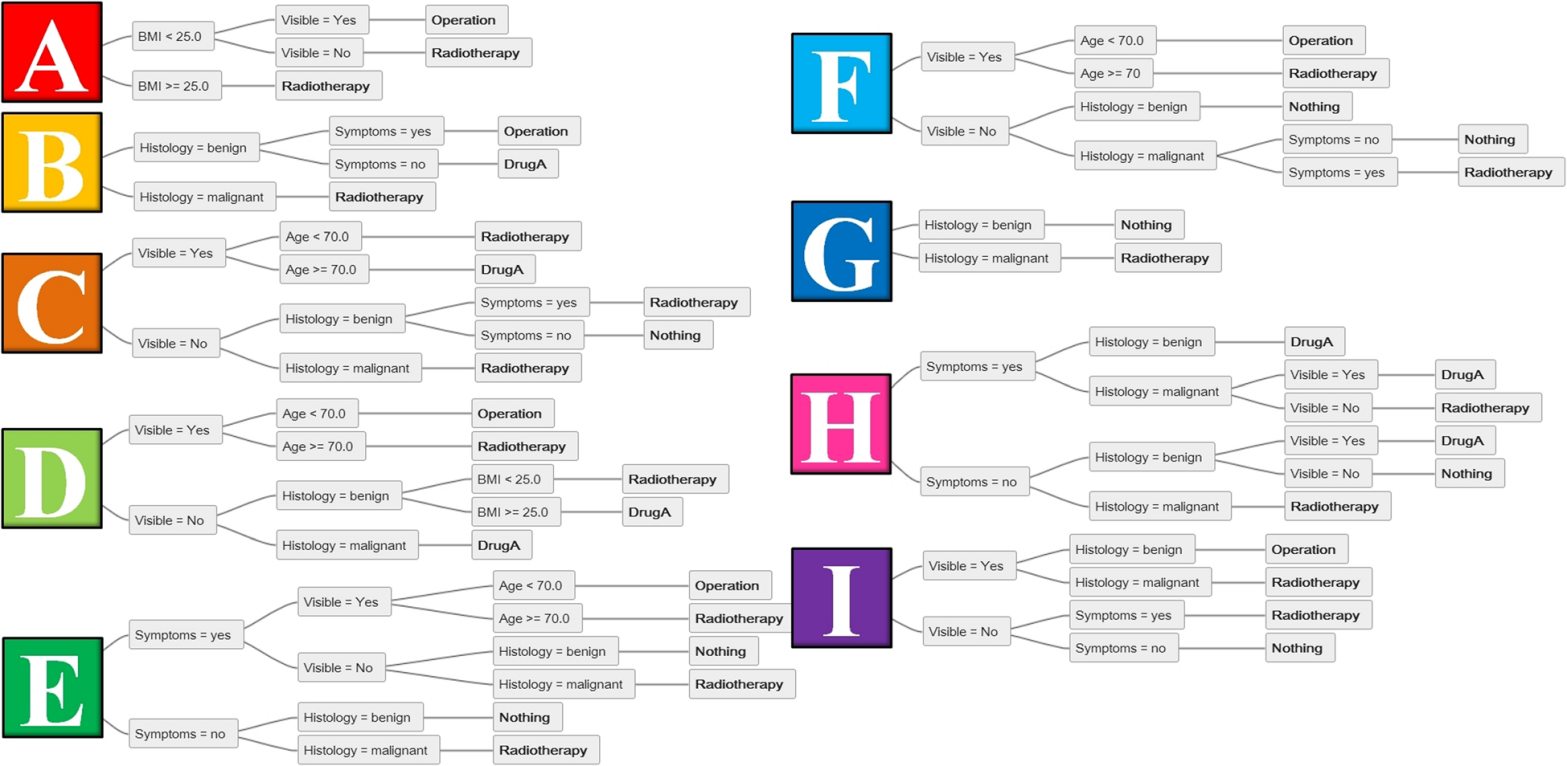
**Nine sample decision trees are named “A” to “I”.** Each tree starts at the root on the left side of the tree. Based on the parameters and their values it is possible to follow each decision tree from left to right to reach a specific treatment recommendation. The recommendations are “Nothing”, “Drug A”, “Radiotherapy” and “Operation” in these examples.

For exploration, simple random criteria were defined (e.g. age, visibility, histology) to include different data types and ranges, i.e. numeric values with a range (age: 0–140 years), Boolean (visibility: true/false) or categorical (histology: benign/malignant). These parameters were randomly combined to create nine different decision trees of varying complexity [17] (Figure 1). To provide altering treatment recommendations “radiotherapy”, “operation”, “DrugA” and “nothing” were randomly assigned to the decision tree branches.

**Figure 2:**
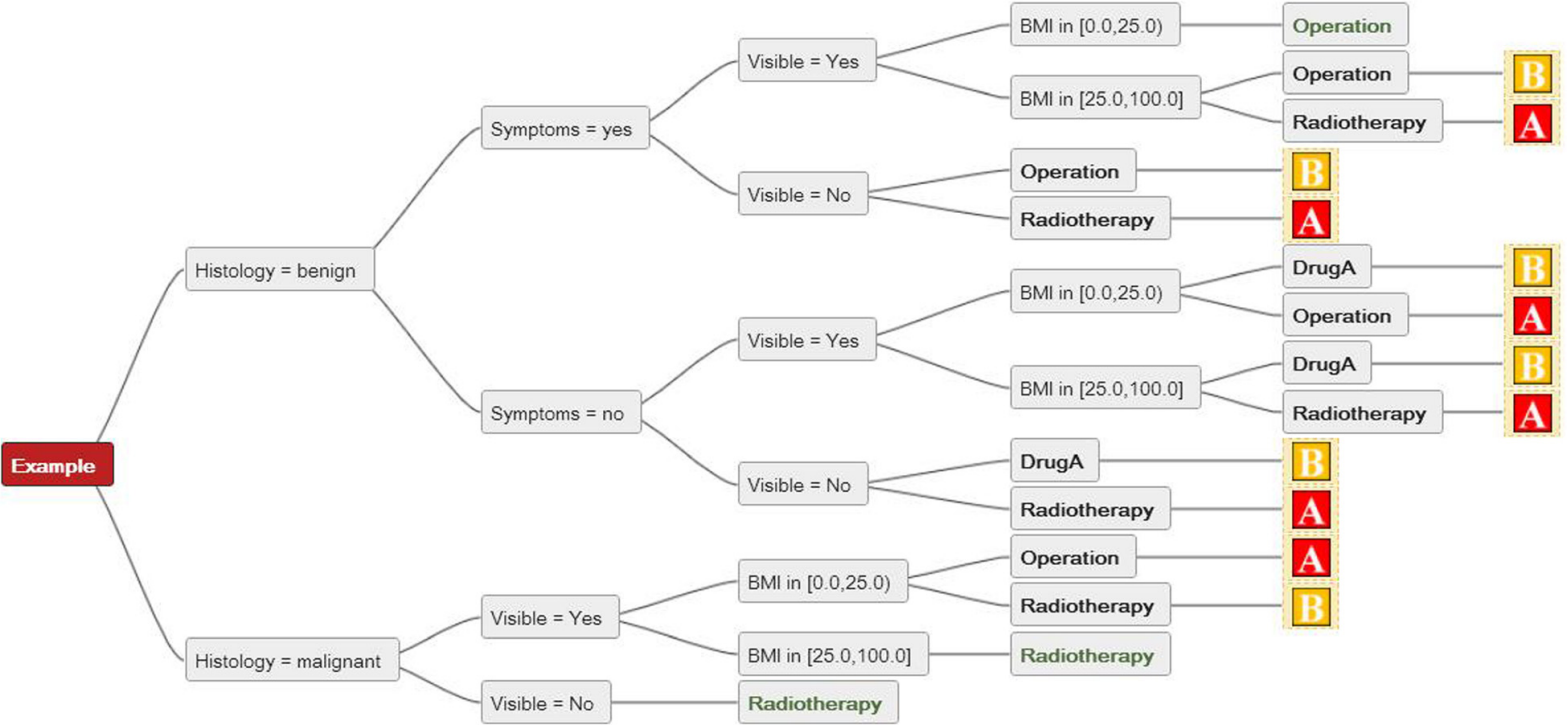
**The result of a direct comparison of two decision trees is displayed.** The resulting tree has a more complex structure than the input trees. When decision trees “A” and “B” are compared, the parameters can be followed from the left to the right to reach specific recommendations for every combination from each party. In the second combination of parameters seen from the top of the graph, party A would recommend an “Operation” whereas party B would recommend “Radiotherapy”. In the bottom row, both parties would recommend “Radiotherapy”.

For any given combination of parameters each tree can be traced from the starting node (left side in the figures) to the final recommendation (leaf). Even if no common parameters are used, any combination of parameters can be tested. For example when the situation “Visible = Yes, BMI < 25 and Histology = malignant” is used (Figures 1 and 2), tree “A” recommends “Operation” and tree “B” “Radiotherapy”. As tree “A” does not implement the parameter “Histology”, this is ignored and the recommendation is based on the other two parameters: “Operation”. In tree “B”, only the parameter “Histology = malignant” is relevant, resulting in “Radiotherapy”.

Analogous to this procedure, the recommendations can be evaluated by considering every possible parameter combination of every decision tree.

To provide a clinical example, we collected and anonymized treatment recommendations on prostate cancer from 16 radiation oncology centres. The prescribed doses for radiotherapy depending on the T-stage, Gleason Score and PSA were collected. Additionally, we collected the criteria for starting androgen deprivation therapy as well as its duration. Only recommendations outside of clinical trials were considered; details on radiotherapy treatment such as margins, patient setup were not considered for this example. Obtaining the decision trees from individuals in various centres consisted of a first query on the general treatment strategy for prostate cancer. These were then specified in brief discussions (mostly per email) and converted into decision trees by PMP and CP. The decision trees were provided to the participating individuals for correction and approval. As the parameters used were identical among centres, the recommendations were converted into a decision tree format using these same parameters.

The decision trees were then analysed to determine the most common recommendations for each possible combination of parameters, based on this, the most common (mode) recommendation could be determined.

The analysis was performed semi-automatically with a web-based software specifically designed for this task. The software was developed in Java programming language using a BigTable database and ran on the Google Cloud Platform AppEngine. Input was provided manually through a website interface which was built using Google Web Toolkit Framework. For the visualisation of decision trees a free JavaScript library called JIT InfoVis has been used. In order to provide comparison results for many and big decision trees in reasonable time the calculation was parallelised and executed on multiple machines within the Google Cloud Platform. For this purpose a mechanism called Task Queue and Google Pipeline application programming interface (API) was used.

### Ethics and consent

The presented research did not involve human subjects, material or data.

## Results

**Figure 3:**
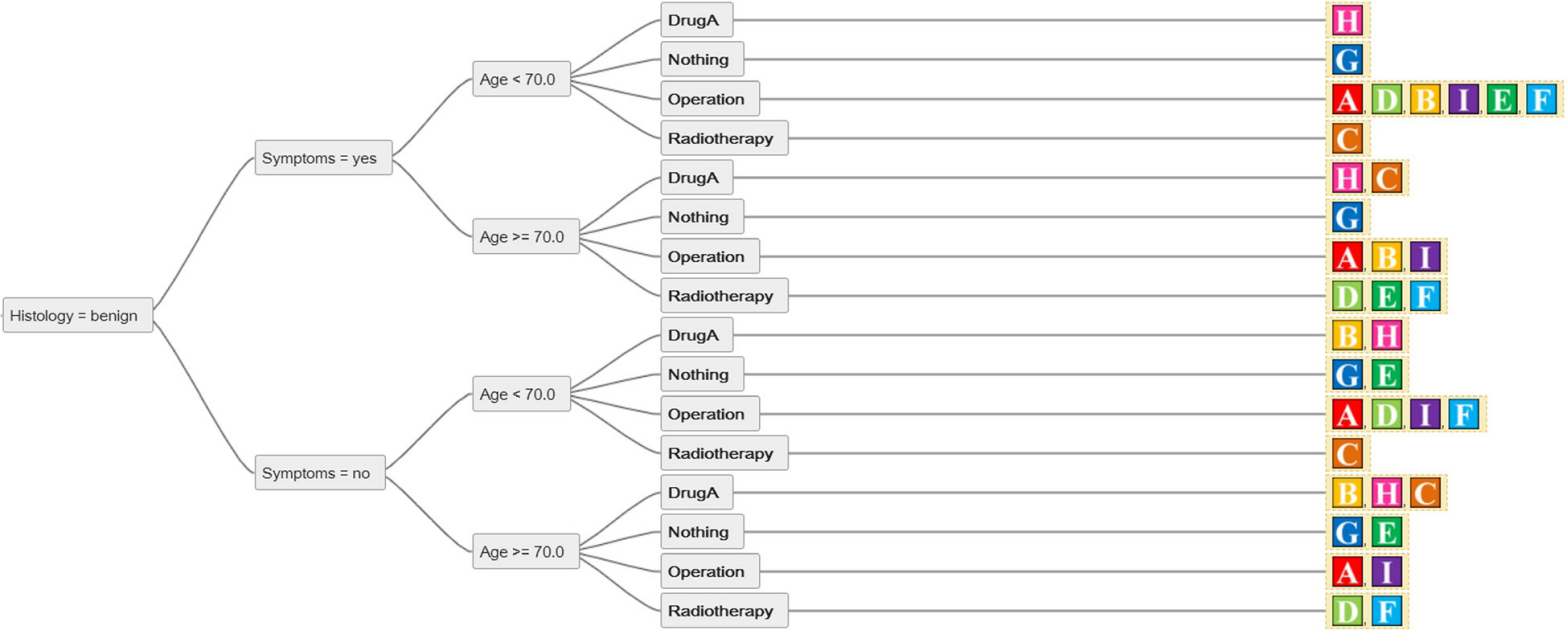
**A partial view of the direct comparison of nine decision trees.** The recommendations of multiple parties are displayed in one tree. In the configuration of parameters on the top of the combined decision tree, all parties except for “H”, “G” and “C” would recommend the treatment option “Operation”.

By a direct comparison of two decision trees the parameters of both are implemented in the result. The combination of decision trees “A” and “B” from Figure 1 results in a more complex structure (Figure 2). Even though decision trees A and B are easily understood by themselves, the complexity of their comparison is less intuitive. The resulting comparison of recommendations of all nine decision trees from Figure 1 is shown in Figure 3.

**Figure 4:**
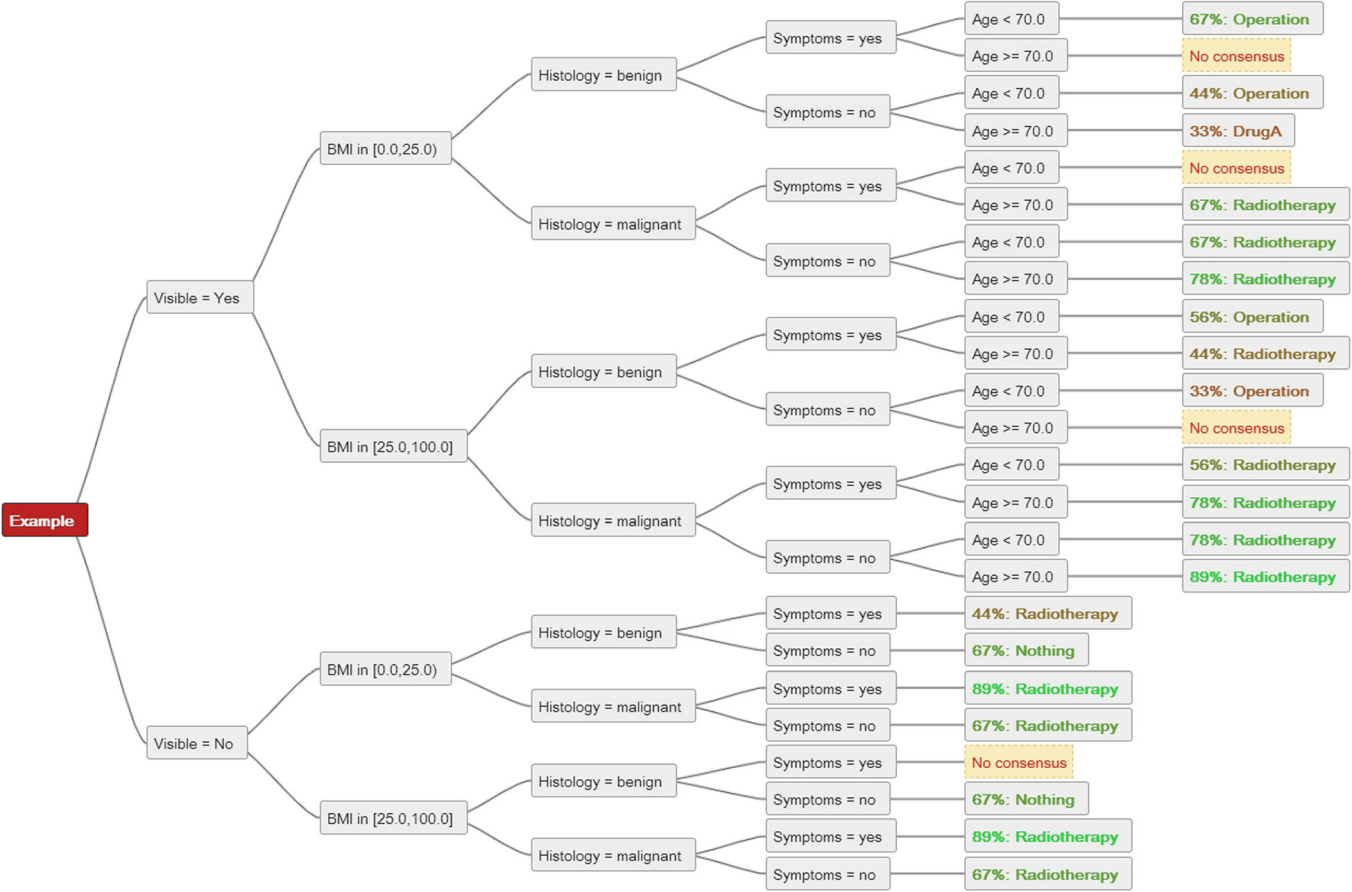
**The mode consensus for 9 input trees is displayed.** All criteria relevant for recommendations are displayed and any parameter combination followed to reach the recommendations on the right. For the top row, when the appropriate criteria are fulfilled the most common recommendation (consensus) was “Operation” for six out of nine recommendation trees (6/9). In the second row, a consensus could not be established (no single dominant mode recommendation for this specific subset).

When all eventualities are analysed, the recommendations can be used as a basis for determining the most common (mode) recommendation for each eventuality separately. These can be used to construct a mode decision tree. In the provided example, consensus was considered to be available if any recommendation was provided as the most common. When two recommendations were equally represented (e.g. three centres “Drug A”, three centres “Radiotherapy”), this was considered no consensus. Where consensus was established, the percentage of congruence was also determined. Figure 4 shows the resulting mode decision tree of nine sample decision trees.

**Figure 5:**
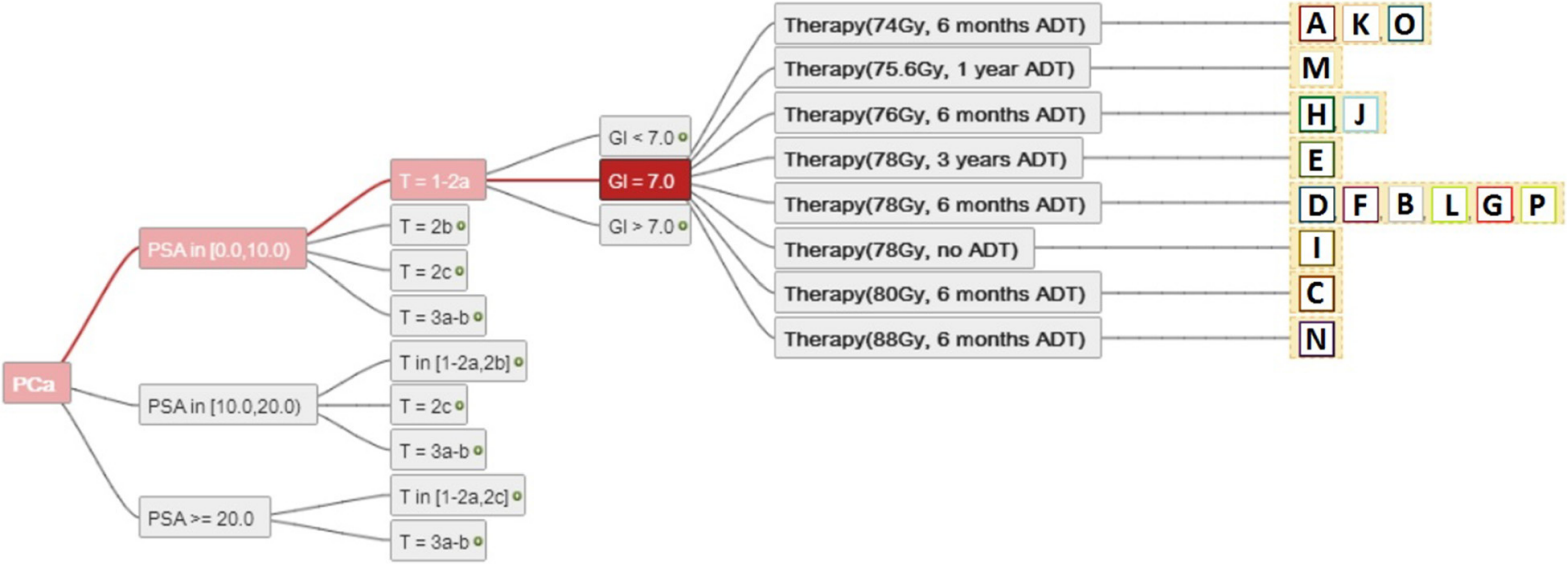
**Recommendations from 16 centres on prostate cancer radiotherapy and androgen deprivation therapy are represented here.** The treatment consists of the prescribed total biologically equivalent dose of RT to the prostate and the duration represents the duration of recommended ADT. Here all recommendations for T stage T1-T2a, PSA < 10 ng/ml and Gleason 7 are represented.

In our clinical example of 16 radiation oncology departments all centres used the same cut-off values for Gleason score, PSA and T-Stage, each divided into three risk groups. This resulted in a maximum of 27 permutations. Based on a direct comparison between these algorithms, all recommendations for the subset of prostate cancer with T stage up to T2a, PSA under 10 ng/ml and a Gleason of 7 are represented (Figure 5).

**Figure 6:**
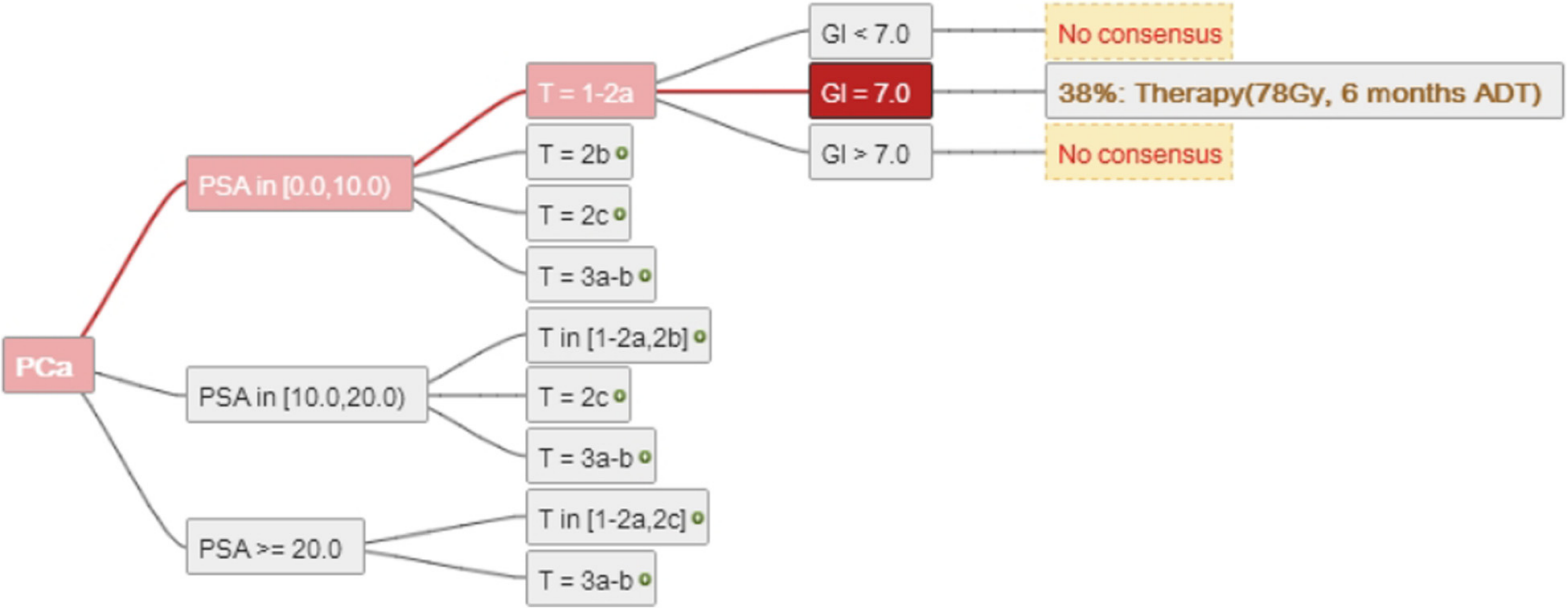
**The consensus tree demonstrates that there is very little consensus in this scenario.** In the scenario with “Gleason = 7” a “consensus” is represented as the most common recommendation. For different Gleason score values no single most common recommendation could be found.

When the direct comparison of the trees was analysed, it was possible to determine whether a mode recommendation was present, and how frequent this was. Figure 6 shows a section of the mode-decision tree, this is derived from an analysis of the full comparison, it is represented by a subset mode recommendation shown in Figure 5.

## Discussion

In order to determine the level of consensus among multiple parties, the individual positions of each party need to be available. In order to provide an objective basis for analysis, a collection of these recommendations in compatible format is required. The ideal format is a complete decision tree representing every possible combination of relevant parameters. However, this is often not readily available. Alternatively a short free-text version can be provided, the essence of which can be transformed into a decision tree. This decision-tree can then be iteratively checked against clinical scenarios and every possible combination of parameters. The parameters need to be clearly defined and agreed upon by all participating parties.

Of note, the implementation of each parameter within a recommendation tree is not mandatory. For instance, the parameter “histology” is not included in tree A but is implemented in others (Figure 1). In clinical routine not every parameter is used by all parties. Due to the inherent structure of a decision tree, the order of the parameters is not relevant as long as the parameter combinations lead to the same recommendation.

The mode decision tree is a transparent method to determine the agreement of multiple recommendations of varying structure within the same clinical context based on objective and standardized interpretation.

Areas of controversy and consensus can be equally represented. The completeness of the mode decision tree can provide users with guidance where traditional consensus methodologies or statements remain inconclusive.

Depending on the context, the anonymity of parties within a consensus effort may be of great value. Analysis of anonymously provided input can exclude any bias towards more influential parties and avoids direct confrontation among parties.

With traditional consensus-finding methods the effort per party increases with the number of parties [1,9], while with the mode-decision tree approach the effort per party is constant irrespective of the total number of participants.

Difficulty in implementing this methodology may result from the effort required to produce a recommendation tree covering all eventualities. Parties are faced with the problem of externalising intrinsic knowledge and every-day know-how in the form of a decision tree [18]. In clinical practice, selected permutations (eventualities) may be very rare and physicians may never have to decide on certain issues. For example, the choice of chemotherapy for lung cancer in a 55-year old pregnant woman with renal insufficiency may never be needed. When recommendations are collected in decision tree format, the users must either provide a recommendation for these situations (as all permutations should be covered) or actively decide that they cannot. Interdependent hierarchical parameters are not suitable for automated decision tree comparison with this method. For example, should a decision tree include the recommendation “operation” and then further recommendations based on how this treatment worked “follow-up” after “gross total resection” or “adjuvant radiotherapy” after “subtotal resection” inconsistencies would arise. The latter recommendations are exclusive to their higher level criteria and not applicable without this condition. Depending on the complexity a potential approach might be to define one decision tree up to the recommendation (e.g. “operation”) and another one with this recommendation as a starting point.

An interesting option is party reselection: creating a mode decision tree from a subgroup (e.g. analysing the input from all participants of a single country). For individual questions, weighting may be based on properties of the parties (e.g. the number of patients treated per party). For example should all users within a set provide their recommendation trees and the number of patients treated, an estimate of how the majority of patients are treated can be made.

Digital communication may not replace direct contact where required, but may help determine various issues before any face-to-face meeting. Dynamic interpretation of up-to-date input allows for an automatic update; the mode decision tree can be instantly re-evaluated from current data.

A limitation of this approach is that its advantages become apparent only at a certain complexity range. Should the issue under evaluation involve e.g. two parameters and few users, a simple table would provide adequate visualisation. In the other extreme, should the context require many complex parameters the number of permutations may exceed millions. The methodology remains applicable, but the result may be too complex for practical interpretation. Feasibility is dependent on the question being asked, should the question involve the search for specific parameter combinations with complete consensus or a specific recommendation – the number of parameters may be higher and still result in a usable product (when filtered for complete consensus for example). Independent of the number of parameters, the system could be used to find the most common recommendation for a specific parameter combination. Typically, to provide feasible output for all parameter combinations the number of parameters should be kept in single digits.

Due to the accepted cancer classification (i.e. TNM-staging system), oncological diseases are very suitable to automated classifications. As demonstrated in the clinical example, when criteria are accepted among parties, it is possible to determine the specific recommendation from each party based on the decision trees (e.g. for a cT2b Gleason 8 prostate cancer with a PSA value of 15.2 ng/ml). The most common recommendation for such a patient can be determined from the mode decision tree. Besides having criteria multiple parties agree upon (e.g. the TNM staging system) it is important to have explicit and complete local standards in place to make any automated comparison feasible in routine practice. The authors are currently involved in projects testing this method in several clinical scenarios with various partners. Once a system of criteria is set up for a specific clinical problem and tested, adding further individuals/centres is associated with relatively little effort, such an approach may form the basis for possible routine clinical implementation. The results of the decision tree analysis, for example the mode decision tree may readily serve as a clinical decision making tool.

If the decision criteria implemented are identical to the criteria used in published guidelines an automated comparison of an individual tree or the mode decision tree to these guidelines would be possible. If this is the aim, parameters should be prospectively defined as it is possible that further criteria might be used in individual trees not considered in published guidelines.

The mode consensus does not provide any reasoning or justifications and should be interpreted for what it is – an objective analysis of the information provided by participants.

## Conclusions

Diagnostic nodes can be used as a basis for a consensus-finding process from different recommendations within the same clinical context. The mode decision tree methodology may provide a useful instrument to enhance existing methodologies in consensus finding and is not limited to specific areas of clinical medicine. We could demonstrate the applicability on an abstract as well as a specific clinical example. As the mode decision trees represent an objective consensus based on current input, it may provide a valuable source of guidance on a case-by-case basis and thus be implemented in daily routine.
